# Supplementary material for: Fatigue following type 2 diabetes: Psychometric testing of the Indonesian version of the multidimensional fatigue Inventory-20 and unmet fatigue-related needs
Source: PLoS One. 2022 Nov 28;17(11):e0278165. doi: 10.1371/journal.pone.0278165 (PMC9704682; doi:10.1371/journal.pone.0278165)
Supplement: S2 Table — (DOCX) [file pone.0278165.s002.docx]

S2 Table. Floor and ceiling effects and internal consistency of IMFI-20

| IMFI-20 | M + SD | Range (min-max) | % Floor effect^a^ | % Ceiling effect^b^ | Cronbachα |
| --- | --- | --- | --- | --- | --- |
| Total score of IMFI-20 | 46.1 ± 13.7 | 20 – 76 | 1.5 | 3.0 | 0.92 |
| General/physical fatigue | 20.6 ± 7.7 | 8 – 40 | 7.5 | 3.0 | 0.92 |
| Mental fatigue | 10.2 ± 3.6 | 4 – 20 | 9.0 | 2.5 | 0.82 |
| Reduced activity | 8.1 ± 3.3 | 4 -16 | 24.0 | 5.0 | 0.85 |
| Reduced motivation | 7.3 ± 2.5 | 4 - 17 | 23.0 | 0.5 | 0.75 |

M + SD, mean ± standard deviations, a, refers to the proportion having the minimum score, b, refers to the proportion having the maximum score.
