# Supplementary material for: Fatigue following type 2 diabetes: Psychometric testing of the Indonesian version of the multidimensional fatigue Inventory-20 and unmet fatigue-related needs
Source: PLoS One. 2022 Nov 28;17(11):e0278165. doi: 10.1371/journal.pone.0278165 (PMC9704682; doi:10.1371/journal.pone.0278165)
Supplement: S3 Table — (DOCX) [file pone.0278165.s003.docx]

S3 Table. Correlations of IMFI-20 with FACIT-F, BDI-II, and PSQI

| IMFI-20 | **FACIT-F** | **BDI-II** | **PSQI** |
| --- | --- | --- | --- |
| Total | -0.71^*^ | 0.67^*^ | 0.58^*^ |
| General and physical fatigue | -0.68^*^ | 0.65^*^ | 0.51^*^ |
| Mental fatigue | -0.43^*^ | 0.38^*^ | 0.35^*^ |
| Reduced activity | -0.65^*^ | 0.61^*^ | 0.55^*^ |
| Reduced motivation | -0.33^*^ | 0.32^*^ | 0.37^*^ |

*P < 0.01. Abbreviations: FACIT-F, The Functional Assessment Chronic Illness Therapy-Fatigue, BDI-II, The Beck Depression Inventory-II, PSQI, The Pittsburgh Sleep Quality Index
